# Vick’s Floral Guide, 1892

**Published:** 1892-03

**Authors:** 


Vick's Floral Guide, 1892.

True and tried friends are always welcome, consequently "Vick's Floral Guide" is sure of a warm reception, especially when dressed as daintily as this year. The "Nellie Lewis" Carnation on the front of cover, and "Brilliant Poppies" on the back, are unusually attractive, and the numerous colored plates of flowers and vegetables are certainly works of art and merit. The first twenty-four pages, printed in violet ink, describe Novelties and Specialties. Send ten cents to James Vick's Sons, Rochester, N. Y., and procure a copy of this attractive and useful catalogue. It costs nothing, as the ten cents can be deducted from the first order.

